# Increased resting state functional connectivity in the fronto-parietal and default mode network in anorexia nervosa

**DOI:** 10.3389/fnbeh.2014.00346

**Published:** 2014-10-02

**Authors:** Ilka Boehm, Daniel Geisler, Joseph A. King, Franziska Ritschel, Maria Seidel, Yacila Deza Araujo, Juliane Petermann, Heidi Lohmeier, Jessika Weiss, Martin Walter, Veit Roessner, Stefan Ehrlich

**Affiliations:** ^1^Department of Child and Adolescent Psychiatry, Translational Developmental Neuroscience Section, Eating Disorder Services and Research Center, Faculty of Medicine, University Hospital C. G. Carus, Technische Universität DresdenDresden, Germany; ^2^Department of Psychotherapy and Psychosomatic Medicine, Faculty of Medicine, University Hospital C. G. Carus, Technische Universität DresdenDresden, Germany; ^3^Department of Psychiatry, Otto-von-Guericke UniversityMagdeburg, Germany; ^4^MGH/MIT/HMS Martinos Center for Biomedical Imaging, Massachusetts General HospitalCharlestown, MA, USA; ^5^Department of Psychiatry, Harvard Medical School, Massachusetts General HospitalBoston, MA, USA

**Keywords:** anorexia nervosa, fMRI, resting state connectivity, fronto-parietal network, default mode network, insula, cognitive control, interoceptive awareness

## Abstract

The etiology of anorexia nervosa (AN) is poorly understood. Results from functional brain imaging studies investigating the neural profile of AN using cognitive and emotional task paradigms are difficult to reconcile. Task-related imaging studies often require a high level of compliance and can only partially explore the distributed nature and complexity of brain function. In this study, resting state functional connectivity imaging was used to investigate well-characterized brain networks potentially relevant to understand the neural mechanisms underlying the symptomatology and etiology of AN. Resting state functional magnetic resonance imaging data was obtained from 35 unmedicated female acute AN patients and 35 closely matched healthy controls female participants (HC) and decomposed using spatial group independent component analyses (ICA). Using validated templates, we identified components covering the fronto-parietal “control” network, the default mode network (DMN), the salience network, the visual and the sensory-motor network. Group comparison revealed an increased functional connectivity between the angular gyrus and the other parts of the fronto-parietal network in patients with AN in comparison to HC. Connectivity of the angular gyrus was positively associated with self-reported persistence in HC. In the DMN, AN patients also showed an increased functional connectivity strength in the anterior insula in comparison to HC. Anterior insula connectivity was associated with self-reported problems with interoceptive awareness. This study, with one of the largest sample to date, shows that acute AN is associated with abnormal brain connectivity in two major resting state networks (RSN). The finding of an increased functional connectivity in the fronto-parietal network adds novel support for the notion of AN as a disorder of excessive cognitive control, whereas the elevated functional connectivity of the anterior insula with the DMN may reflect the high levels of self- and body-focused ruminations when AN patients are at rest.

## Introduction

Anorexia nervosa (AN) is characterized by a disturbed body image, constant preoccupations with weight and shape and an intensive fear of weight gain that lead to severe dietary restriction and weight loss (American Psychiatric Association, 2013). Additional common symptoms include perfectionism, excessive physical activity, alexithymia and disturbed interoceptive awareness (Lilenfeld et al., 2006).

Given the variety of AN symptoms, different research approaches and hypotheses have been pursued in the last decade. For example, some researchers have focused on executive functioning and volition and have described AN as a disorder of enhanced higher-order cognitive control functions (Kaye et al., 2009, 2013; Zastrow et al., 2009). Others have targeted the presumably impaired processing of rewarding and emotional stimuli (Wagner et al., 2007b; Brooks et al., 2011; Oberndorfer et al., 2011; Frank et al., 2012; Bischoff-Grethe et al., 2013). Additional major areas of AN research include the distorted body image (Gaudio and Quattrocchi, 2012), deficits in interoceptive awareness and excessive rumination about body shape and food intake (Fassino et al., 2004; Friederich et al., 2010; Sternheim et al., 2012). Given these diverse research approaches and first results, a neurobiological framework of AN based on dorsal and ventral neurocircuit dysfunctions has been proposed (Kaye et al., 2009, 2013). Initially, this framework was used to explain symptoms of schizophrenia, bipolar disorder and major depression (Phillips et al., 2003b). The ventral limbic circuit which encompasses amongst others amygdala, insula, ventral striatum, and ventral medial cortex identifies emotional significance and contributes to affective states. In contrast, the dorsal executive neurocircuit, including brain regions such as dorsal regions of the ACC, dorsolateral prefrontal cortex and parietal cortex, is important for selective attention, planning and effortful regulation of affective states (Phillips et al., 2003a).

Neuroimaging studies in AN provide evidence for altered (often decreased) neural responses to rewarding stimuli in limbic brain regions (Wagner et al., 2007b; Oberndorfer et al., 2011) while at the same time neural responses in frontal and parietal brain suggest enhanced inhibitory abilities and increased cognitive control (Zastrow et al., 2009; Favaro et al., 2012). These findings in AN can be interpreted as an imbalance between ventral limbic and dorsal executive brain circuits. However, if compared to other neuropsychiatric disorders the application of this framework to AN rests on a rather small number of mostly task-based neuroimaging studies. Task-based neuroimaging studies have the advantage that they may be able to target specific functions or neurocircuits. However, the usage of different designs and stimuli makes it difficult to compare and reproduce such studies. Furthermore, it may vary how patients understand the task and which strategy they use or whether their response is biased by social desirability (Damoiseaux et al., 2006; Salbach-Andrae et al., 2008). Therefore, an additional research approach that allows the assessment of task-independent intrinsic networks might be useful to circumvent the aforementioned obstacles and help to complement the knowledge generated using task-based neuroimaging studies.

Resting state functional connectivity (rs-FC) is a relatively novel approach to study synchronous low frequency blood oxygen level-dependent (BOLD) signal fluctuation of brain regions recorded during rest to define brain networks at a macroscopic level (Fox and Raichle, 2007). Brain networks that are based on temporally correlated intrinsic fluctuation of spatially distinct and functionally highly relevant brain regions are relatively stable across subjects and have been termed resting state networks (RSN). rs-FC constitutes a particular suitable approach for clinical studies as it does not require much cooperation of the often burdened patients. Moreover, it allows investigating aberrant connectivity which has been proposed to be a core feature of psychiatric disorders (Menon, 2011). One major difference to task fMRI studies arises from the possibility to investigate local neural responses and interregional connections for all brain regions within the same experimental protocol. This advantage clearly adds to a better understanding of complex brain disorders, where tasks addressing all regions homogeneously are not feasible and combination of different task settings is limited due to time constraints. In contrast, resting state acquisitions are normally acquired within 5–10 min.

A limited number of studies have investigated RSNs in patients with AN. So far, results have been somewhat heterogeneous. Cowdrey et al. (2014) reported increased functional connectivity in the well-defined default mode network (DMN), encompassing the ventral-medial prefrontal cortex, precuneus, inferior parietal lobule and lateral parts of the temporal cortex, in patients recovered from AN whereas normal functional connectivity was reported for the visual, somato-sensory and cognitive control network. These findings were challenged by McFadden et al. (2014) who reported reduced functional connectivity in the DMN for patients with AN in comparison to HC. Favaro et al. (2012) found decreased functional connectivity in the ventral visual network, while the opposite was true for the somato-sensory network. Using a seed-based approach Favaro et al. (2014) described decreased functional connectivity of the dorsal putamen in patients with AN. In order to gain a deeper understanding of functional connectivity in AN further studies systematically investigating AN-relevant RSNs in larger and more homogenous samples are needed.

Here we used independent component analyses (ICA), a data-based approach, to identify RSNs in resting state functional magnetic resonance imaging (fMRI) data of a relatively large homogenous, unmedicated sample of young acute AN patients and closely matched healthy controls. To capture possible abnormalities of brain networks corresponding to typical AN symptom domains and popular hypotheses on the etiology of AN as introduced above, we focused on the following RSN: the fronto-parietal network to investigate the notion of excessive cognitive control, the salience network to understand the suspected aberrant processing of rewarding and emotional stimuli in AN, the visual and somatosensory networks to uncover possible neural correlates of the body image distortion and the DMN related to self-referential processing including disorder-typical rumination. Based on the current neurobiological framework of AN (Kaye et al., 2009), we expected hyperconnectivity in networks encompassing the dorsal neurocircuit, in particular the fronto-parietal network but hypoconnectivity in the ventral neurocircuit, namely the salience network.

## Methods

### Participants

The sample of the current study consisted of a total of 70 female volunteers: 35 patients with acute AN according to DSM-IV (AN patients; 12–23 years old) and 35 female healthy controls (HC; 12–23 years old). Case-control age-matching was carried out using the Munkres algorithm (Munkres, 1957) resulting in a maximum difference of 0.9 years between the individuals within one pair. All AN patients were admitted to eating disorder programs of a university child and adolescent psychiatry and psychosomatic medicine department and were assessed within 96 h after the beginning of a behaviorally-oriented nutritional rehabilitation program. Within the AN group, 33 (94.3%) of the patients were of the restricitive and 2 (5.7%) of the binge/purging subtype; 4 (11.4%) had comorbid psychiatric disorders (two patients with depressive disorders including dysthymia, one with anxiety disorder and one with obsessive compulsive disorder). HC participants had to be of normal weight and eumenorrhoeic and without any history of psychiatric illness. HCs were recruited through advertisement among middle school, high school and university students.

Exclusion criteria and possible confounding variables for patients with AN, including psychotropic medication, binge eating, or diagnosis of bulimia nervosa, were obtained using a semistructured research interview, the SIAB-EX interview (see below) and our own medical records. Comorbid diagnoses were taken according to standard practice from medical records and confirmed by an expert clinician with over 10 years of experience after careful chart review (including consideration of medical and psychiatric history, physical examination, routine blood tests, urine analysis and a range of psychiatric screening instruments).

HC participants were excluded if they had any history of psychiatric illness, a lifetime body-mass index (BMI) below the 10th age percentile (if younger than 18 years)/BMI below 18.5 kg/m^2^ (if older than 18 years), or were currently obese (BMI not over 97th age percentile if younger than 18 years; BMI not over 30 kg/m^2^ if older than 18 years). Participants of all study groups were excluded if they had a lifetime history of any of the following clinical diagnoses: organic brain syndrome, schizophrenia, substance dependence, psychosis not otherwise specified (NOS), bipolar disorder, bulimia nervosa or binge-eating disorder (or “regular” binge eating—defined as bingeing at least once weekly for 3 or more consecutive months). Further exclusion criteria for all participants were intelligence quotient (IQ) lower than 85; psychotropic medication within 4 weeks prior to the study; current substance abuse; current inflammatory, neurologic or metabolic illness; chronic medical or neurological illness that could affect appetite, eating behavior, or body weight (e.g., diabetes); clinical relevant anemia; pregnancy or breast feeding.

Study data were collected between September 2011 and November 2013 and managed using secure, web-based electronic data capture tools REDCap (Research Electronic Data Capture) (Harris et al., 2009). This study was approved by the local Institutional Review Board, and all participants (and if underage their guardians) gave written informed consent.

### Clinical measures

For all participants, current diagnoses of eating disorders were ascertained by evaluation of the expert form of SIAB-EX (Fichter and Quadflieg, 1999), a well-validated 87-item semi-standardized interview that assesses the prevalence and severity of specific eating-related psychopathology over the past 3 months. The interview provides diagnoses according to the ICD-10 and DSM-IV. Interviews were conducted by clinically experienced and trained research assistants under the supervision of the attending child and adolescent psychiatrist.

Eating disorder-specific psychopathology was assessed with the short version of the Eating Disorders Inventory (EDI-2), a self-report comprising 8 subscales (Paul and Thiel, 2005). Response categories range from 1 “never” to 6 “always”. Given the assumption that AN is characterized by disturbances in the processing of bodily signals and alexithymia (Fassino et al., 2004), we utilized the subscale “interoceptive awareness” as part of a confirmatory analysis (regarding the DMN and somatosensory network) in the current study.

Personality dimensions were assessed using the German version of the Junior temperament and character inventory (JTCI; Goth and Schmeck, 2009) which is based on Cloninger’s biosocial model of personality (Cloninger, 1994). Of interest for our current study (in relation to the fronto-parietal “control” and salience network respectively) are the temperament dimensions “persistence” and “reward-dependence” as patients with AN are believed to exert excessive cognitive control and impaired reward processing (Friederich et al., 2013).

IQ was measured with a short version (including the subtests: picture completion, digit symbol-coding, similarities and arithmetic) of the German adaption of the Wechsler Adult Intelligence Scale (WIE; von Aster et al., 2006) for participants aged 16 years and older or a short version (including the subtests: vocabulary, letter-number-sequencing, matrix reasoning and symbol search) of the German adaption of the Wechsler Intelligence Scale for Children (HAWIK; Daseking et al., 2007) for participants aged 15 years or younger.

### Data acquisition

Data was acquired with a 3T Siemens Trio (UMN). The T1-weighted structural brain scans were acquired with rapid acquisition gradient echo (MP-RAGE) sequence with the following parameters: number of slices = 176; repetition time = 1900 ms; echo time = 2.26 ms; flip angle = 9°; slice thickness = 1 mm; voxel size = 1 × 1 × 1 mm^3^; field-of-view = 256 × 224 mm^2^; bandwidth = 2004 Hz/pixel.

The functional images were acquired by using a gradient-echo T2*-weighted echo planar imaging (EPI) with the following parameters: tilted 30° towards AC–PC line (to reduce signal dropout in orbitofrontal regions); number of volumes = 190; number of slices = 40; repetition time = 2200 ms; echo time = 30 ms; flip angle (FA) of 75°; 3.4 mm in-plane resolution; slice thickness of 2.4 mm (1 mm gap resulting in a voxel size of 3.4 × 3.4 × 2.4 mm^3^); FoV = 220 × 220 mm^2^; bandwidth of 200 Hz/pixel. During fMRI participants were instructed to lie still with closed eyes and without falling asleep.

### Image data preprocessing

Functional and structural images were processed using SPM8 toolbox^1^ within the Nipype framework^2^ (Gorgolewski et al., 2011). The slice time corrected functional data were realigned and registered to their mean. The realigned files were coregistered to the subject’s structural brain image. A DARTEL template was created using structural images from all subjects (Ashburner, 2007). The EPI volumes were then normalized to MNI space using the DARTEL template and corresponding flow field. The resulting data were smoothed with an isotropic 8 mm FWHM Gaussian kernel.

We evaluated the quality of the fMRI data by manual inspection and using artifact detection tools (ART; Whitfield-Gabrieli et al., 2009). Volumes that exceed an intensity threshold of three standard deviations or a threshold of 2 mm normalized movement in any direction were classified as outliers (motion-outlier: AN patients: 0.31 ± 0.87, HC: 0.97 ± 2.67; intensity-outlier: AN patients: 1.5 ± 2.37, HC: 2.38 ± 2.85); the two groups did not differ regarding numbers of motion- and intensity-outliers (motion-outlier: *t*_(68)_ = 1.38, *p* = 0.17; intensity-outlier: *t*_(68)_ = 1.38, *p* = 0.17).

### Independent component analysis

To identify temporally coherent RSNs we conducted a spatial group ICA for all 70 participants (Calhoun et al., 2001) using the Group ICA fMRI Toolbox (GIFT) implemented in Matlab.^3^ The fMRI data was decomposed into maximally independent components according to the following steps: The number of components were estimated using minimum description length criteria, modified to account for spatial correlation (Li et al., 2007). Due to computational feasibility the previously concatenated data from all subjects was reduced using Principal Component Analysis (PCA). An ICA using the infomax algorithm was then applied to the data (Bell and Sejnowski, 1995). For each subject, component spatial maps were reconstructed (back-reconstruction using GICA) and converted to *z*-values.

### Component selection

To identify components of interest for further analysis a systematic two-step process was applied (Assaf et al., 2010; Kullmann et al., 2012). First, all components likely to be artifacts were identified by correlating the component’s spatial maps with a priori mask of white matter and cerebral spinal fluid (CSF) (MNI template provided in SPM 8). Components correlating significantly with white matter and/or CSF were identified as artifacts and subsequently removed from the analysis. Second, the remaining components were then spatially correlated with specific RSN templates obtained in over 1000 healthy subjects by Yeo et al. (2011) to identify components covering the frontal-parietal network, the DMN, the salience network, the visual and the sensory-motor network. Components with a significant correlation (*p* < 0.05, two-tailed) with a RSN template were selected for further analyses. In case of significant overlap with two templates, the RSN with the highest correlation was assigned.

### Statistical analyses

For group analyses, spatial maps of the back-reconstructed components representing the networks of interest were entered into SPM8. *Z*-values of these maps represent the concordance of the voxel-specific time-course to the averaged components time-course. Thus, a group comparison of the spatial maps reflects a group difference in the connectivity strength or signal synchronization of each voxel to the whole spatial component. To examine group differences a two-sample *t*-test was performed for each preselected component. The resulting statistical maps were masked with the aforementioned RSN templates (Yeo et al., 2011). Between group differences had to exceed *p* < 0.05 family-wise error (FWE; at the cluster-level) to guard against type I errors.

In order to further assess the associations of the magnitude of group differences in rs-FC with psychometric parameters we extracted the beta values of the respective clusters at a threshold of *p* < 0.001 (uncorrected) using Marsbar (Brett et al., 2002) to account for greater anatomical variability and computed Pearson’s *r* with SPSS statistical software version 21.0 (SPSS, Chicao, Illinois) separately for each subgroup. Based on previous literature and the assumed functionality of the RSN we selected specific self-report measures a priori to be tested for associations with each RSN, i.e., persistence (JTCI) for the frontal-parietal RSN, interoception (EDI-2) for the DMN and body dissatisfaction (EDI-2) for visual and somato-sensory RSN. To address possible developmental effects we also investigated the association between age and the magnitude of rs-FC using the same approach.

## Results

### Participants

As shown in Table 1, there were no significant group differences for age and IQ. As expected, AN patients had a significantly lower BMI, a significantly higher EDI-2 total score, interoceptive awareness score as well as persistence score than HC.

**Table 1:** **Sociodemographic and clinical variables of the two groups; BMI = Body mass index, IQ = intelligence quotient, EDI-2 = eating disorder inventory, JTCI = Junior Temperament and Character Inventory, BMI and minimal lifetime BMI are displayed but statistical comparisons are based on BMI-SDS values to ensure comparability across age, IQ was assessed with a short version of the German adaption of the Wechsler Adult Intelligence Scale (von Aster et al., 2006) for participants aged 16 years and older or a short version of the German adaption of the Wechsler Intelligence Scale for Children (Petermann and Petermann, 2008) for participants aged 15 years or younger**.

	AN (*n* = 35)	HC (*n* = 35)	*t*	*p*
**Demographic variables**
Age	16.10 ± 2.56	16.16 ± 2.64	−0.09	0.93
BMI	14.78 ± 1.26	20.81 ± 2.72	−11.9	>0.000
BMI-SDS	−3.16 ± 1.43	0.01 ± 0.83	−11.4	>0.000
IQ	111.09 ± 11.47	112.03 ± 9.79	−0.36	0.72
**Clinical variables**
Age of onset	13.50 ± 1.70	n.a.	–	–
Duration of current AN episode (in month)	18.94 ± 27.06	n.a.	–	–
EDI-2 total	199.19 ± 48.32	143.45 ± 29.27	5.77	>0.000
Interoceptive awareness (EDI-2)	29.16 ± 10.26	18.94 ± 5.94	10.62	>0.000
Persistence (JTCI)	53.35 ± 8.56	46.05 ± 6.98	−3.88	>0.000

*Group differences were tested using Student’s t-tests*.

### Component identification

**Figure 1:**
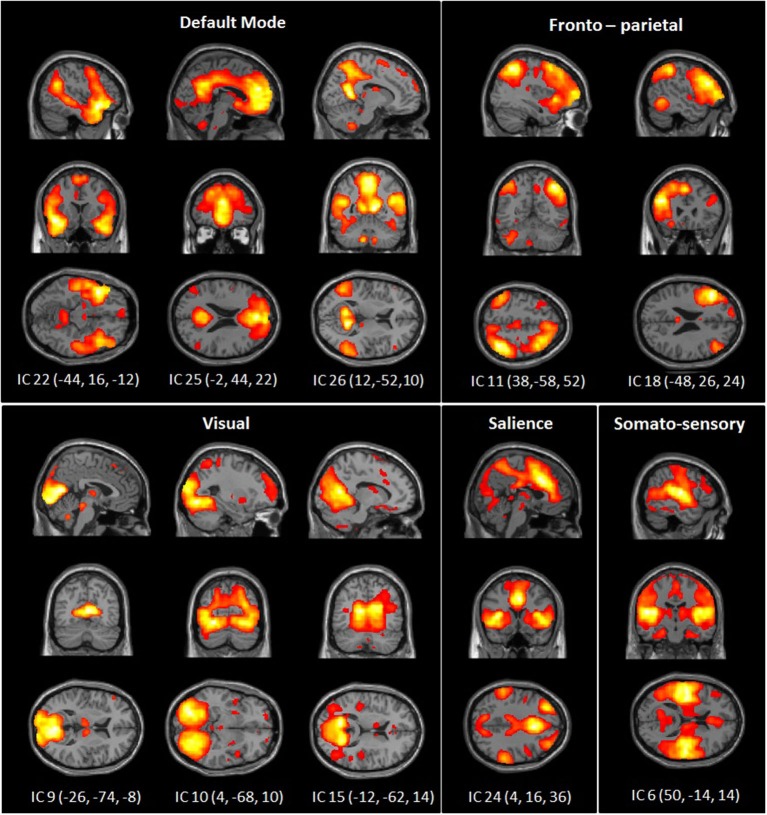
**Spatial maps of 10 independent components of interest grouped by network: DMN, somato-sensory, visual, fronto-parietal and salience network**. Spatial maps are plotted as *t*-statistics thresholded at *p* = 0.05 (FWE).

The dimension estimation of the rs-FC data resulted in 25.53 dimensions (SD = 7.52) on average. The PCA analyses reduced the data set to 39 dimensions and ICA extracted 26 independent components (IC). Of these, 10 components were identified as RSNs of interest (Figure 1) and five components were identified as artifacts (correlating significantly with a priori map of CSF).

### Group comparison

**Figure 2:**
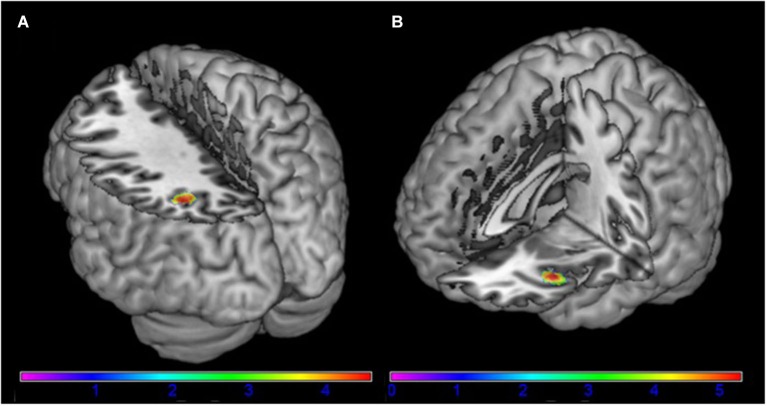
**Differences between patients with acute anorexia nervosa and healthy controls (A) in component 18, representing the frontal-parietal network and (B) in component 24, representing the default-mode network (for illustrative purposes shown at *p* = 0.001 uncorrected)**. Color bar represents *t*-values.

The left angular gyrus showed increased functional connectivity with component 18, associated with the frontal-parietal RSN (*t*_peak_ = 4.58; *p* = 0.047 (FWE) [−34, −60, 38]) in AN patients in comparison to HC (Figure 2). Furthermore, we found a significantly increased functional connectivity between component 26 associated with the DMN and the left anterior insula/frontal operculum (*t*_peak_ = 5.32; *p* = 0.007 (FWE) [−38, 22, −8]) in AN patients in comparison to HC (Figure 2). When controling for comorbid disorders (*n* = 4) or AN subtype (two patients of the binge/purging subtype) the group differences remained significant. No group differences were observed in the remaining components.

### Association of group differences in RSN connectivity with psychometric parameters and age

Individual connectivity values were extracted from clusters that showed significant group differences and correlated with self-report measures corresponding to the selected RSN in each group separately. Extracted beta values of the frontal-parietal network cluster indicated that the subscale persistence of the JTCI was significantly correlated with functional connectivity strength of the angular gyrus in HC (*r* = 0.532; *p* = 0.001) but not in AN patients (*r* = −0.058, *p* = 0.743). Regarding the DMN component 26, we found a significant correlation with problems with interoceptive awareness and the extracted beta values at the anterior insula/frontal operculum for HC (*r* = 0.341, *p* = 0.045) but not for AN patients (*r* = −0.058, *p* = 0.743). Age was neither associated with the functional connectivity in the angular gyrus (*r* = −0.11; *p* = 0.36) nor in the anterior insula/frontal operculum (*r* = −0.15; *p* = 0.20).

## Discussion

In this study we investigated functional connectivity in RSNs which were hypothesized to be relevant to AN symptomatology in a large, homogeneous unmedicated sample of AN patients and closely matched HC. In line with the popular notion to conceptualize AN as a model-disorder for excessive cognitive control (Kaye et al., 2009) we found increased functional connectivity in the fronto-parietal network in patients with AN. However, despite the existing body of research on abnormal reward processing in AN (Frank, 2013) there were no group differences in functional connectivity in the salience network. In contrast, we found the anterior insula, a part of the ventral neurocircuit, to be more strongly connected to the DMN in patients with AN.

Studying rs-FC gave us the opportunity to probe five potentially AN-associated brain networks. Based on our results, abnormalities in the fronto-parietal and DMN network may help to understand AN etiology. Interestingly, for both networks we also found correlations with self-report measures indicating that among healthy controls, those who share some subthreshold features with AN patients also have RSN characteristics that are somewhat more similar to AN. The fact that these associations are present in healthy controls may indicate that these RSN characteristics are expressed along a continuum and might therefore represent a vulnerability factor rather than a consequence of AN. However an alternative explanation may also be that self-report measures are more reliable in HC due to symptom denial in AN patients (Salbach-Andrae et al., 2008).

In detail, the functional connectivity of the angular gyrus with other parts of the fronto-parietal network was positively associated with self-reported persistence in HC. The fronto-parietal network includes the lateral prefrontal cortex and parietal regions (Yeo et al., 2011; Agosta et al., 2012) and has been implicated in a wide range of executive functions, including working memory, performance monitoring and planning. These functions are not restricted to a particular cognitive domain but serve as a general cognitive control process allocating top-down attentional resources to organize cognitive operations (Fassbender et al., 2006; Cole and Schneider, 2007; Niendam et al., 2012).

Results from studies in healthy controls suggest strong relations between cognitive control, eating and weight, e.g., the ability to inhibit responses in a stop signal task was predictive of weight change during a 1-year follow up period (Nederkoorn et al., 2010). Another study used repetitive transcranial magnetic stimulation (rTMS) to modulate frontal brain regions. This led to a marked change in preference ratings for high-caloric food items (Camus et al., 2009). Clinical observations, such as perseverative, obsessive, and rigid thinking styles as well as personality characteristics like low impulsivity and high harm avoidance (Anderluh et al., 2003; Lilenfeld et al., 2006) support the assumption of increased cognitive control in AN patients. In line with these clinical observations neuropsychological studies found reduced cognitive flexibility (e.g., disengagement from previously relevant rules in order to learn new ones) and an excessively detailed information processing (e.g., weak central coherence) (Holliday et al., 2005; Roberts et al., 2007). Task-based neuroimaging studies have also provided evidence suggesting that cognitive control might be elevated in AN. A study by Wagner et al. (2007b) reported a “strategic” response to rewarding stimuli with an increased involvement of prefrontal and parietal cortices, brain regions associated with planning. Using a set-shifting task, Zastrow et al. (2009) found increased activity in a fronto-parietal network which was interpreted as effortful and supervisory cognitive control during task performance. Moreover, two studies using go/no-go tasks provide suggestive evidence for abnormal neural responses in frontal brain regions in AN when behavioral inhibition is required during no-go trials (Lock et al., 2011; Kullmann et al., 2014a).

A recent fMRI study investigating rs-FC in AN using a seed-based approach found increased coupling between parietal regions and the dorsal anterior cingulate cortex in patients with AN, which is also in line with the hypothesis of enhanced higher-order cognitive control functions in AN (Lee et al., 2014). However, a study using more abstract metrics representing whole brain connectivity (degree centrality) and measures of effective connectivity described reduced connectivity within the cognitive control system (Kullmann et al., 2014b), while the only other study employing ICA to target the fronto-parietal network reported no differences between weight-restored patients with AN and HC (Cowdrey et al., 2014). Favaro et al. (2013) found no difference in functional connectivity between acute patients with AN and HC when setting seeds in the dorsolateral, ventrolateral and ventromedial prefrontal cortex. However, in a largely overlapping sample, Favaro et al. (2014) found decreased functional connectivity between the bilateral dorsal putamen, regions that have also been implicated in (habitual) cognitive control (Dolan and Dayan, 2013). One reason for these inconsistencies might be the different data analysis approaches applied in the studies. For example, seed-based approaches are highly dependent on the selected seed region, size of the seed and the applied smoothing (van den Heuvel and Hulshoff Pol, 2010; de Reus and van den Heuvel, 2013). Other reasons for inconsistency might be that patients were in a different stage of the illness, received psychotropic medications or related to the low scanner field strength (resulting in lower spatial resolution) employed in the latter studies. Taken together, some of these studies and our current work are supportive of the view of AN as a neurobiologically-based disorder with increased higher-order inhibitory network function. However the evidence remains still heterogeneous and further studies, e.g., comparing acute and recovered AN patients, are needed.

As mentioned above, we also found an increased functional connectivity in the anterior insula with the DMN in patients with AN. Moreover the functional connectivity in the anterior insula was positively associated with difficulties in interoceptive awareness in HC indicating that HC that have problems with interoceptive awareness also have a DMN functional connectivity that resembles that of AN patients. The self-reported problems with interoceptive awareness include amongst others statements about difficulties to name own feelings.

The DMN is a well-investigated RSN with central hubs in the ventral-medial prefrontal cortex, precuneus, inferior parietal lobule and lateral temporal cortex. Based on known functions of the included brain areas it has been assumed that the DMN supports amongst others internal mentation, self-relevant processing and mentalizing (Buckner et al., 2008). A large number of research reports have emphasized the relevance of the DMN as a key neurobiological system associated with mental disorders such as autism (Kennedy and Courchesne, 2008), schizophrenia (Bluhm et al., 2007) and Alzheimer’s disease (Greicius et al., 2004). Two studies investigating the DMN in AN described also an altered functional connectivity between parietal regions and the DMN although these studies are ambiguous regarding hyper- or hypoconnectivity. In detail, Cowdrey et al. (2011) reported an increased functional connectivity in the DMN in patients recovered from AN whereas McFadden et al. (2014) reported a lower functional connectivity in this network in patients with AN in comparison to HC.

The major group difference in our study was located in the left anterior insula. The insula is a relatively large brain region and studies on functional differentiation of the insula showed that the mid-posterior insula is foremost processing sensory-motor information including exteroceptive information (pain, temperature and itch) and interoceptive information (vasomotor activity, hunger and thirst) whereas the anterior insula has a pivotal role in the downstream processing that integrates these primary representations coming from the mid-posterior insula regions (Craig, 2009; Kurth et al., 2010). In this regard the anterior insula is important in generating emotional states based on the fast and unconscious processing of exteroceptive and interoceptive sensory input. This is supported by studies showing that alexithymia, the inability to identify, analyze and verbalize feelings is associated with dysfunctions of the anterior insula (Bird et al., 2010). A study investigating large-scale brain networks revealed increased connectivity in the insula after watching emotional movies (Eryilmaz et al., 2011) while another study found an association between functional connectivity of the insula with the DMN and anxiety in healthy subjects (Dennis et al., 2011). Taken together, recent research highlights the unique role of the anterior insula in the ability to reflect about one-self or in other words to be aware of one’s own being (Craig, 2009).

According to these ascribed functions of the insula, dysfunctions of this brain region have been suggested to constitute a core factor for the etiology of AN (Nunn et al., 2008). Indeed, a large body of neuroimaging studies in AN have yielded differential neural responses in this region, e.g., during the processing of visual (Brooks et al., 2011; Cowdrey et al., 2011; Holsen et al., 2012; Lawson et al., 2012; Oberndorfer et al., 2013) or actual food stimuli (Wagner et al., 2007a; Frank et al., 2012). Studies employing body image stimuli found consistently a hyperactivation in the insula when patients with AN are confronted with self-images (Friederich et al., 2010; Mohr et al., 2010). These findings are in line with the important role of the insula in AN and the hypothesized function of the insula in body awareness and self-referential processing (Craig, 2009). Interestingly, insular volumes have also been reported to be increased in acute AN (Frank et al., 2013).

Given the fact that AN patients suffer from a distorted sense of self, including symptoms as distorted body awareness and alexithymia (Kaye et al., 2009), the insula-DMN hyperconnectivity observed here can be interpreted in the framework of the triple network model of psychopathology. The triple network model of psychopathology advocates the insula as a central hub of the salience network that mediates the switch from the DMN to the fronto-parietal network as a response to salient stimuli. Once such a salient stimulus is detected the anterior insula disengages the DMN and helps to engage the fronto-parietal network to facilitate task-related executive functioning (Menon, 2011). An increased assignment of the anterior insula to the DMN (instead of the salience network) was also reported by Horn et al. (2010) in patients with major depression. Thus, our finding might reflect the difficulty of AN patients to disengage from an internally oriented mental state when at rest and mirror the high levels of disorder-specific worry and rumination. This seems conclusive, given that patients with major depression report similarly high levels of worry and rumination as patients with AN (Yook et al., 2010). However, patients with AN are typically eager to engage in physically or cognitively demanding tasks, which might be reflected by the heightened fronto-parietal connectivity and serve as a strategy against ruminative and self-focused thoughts.

Importantly, such between network connectivity, as observed for anterior insula and DMN, has gained increasing attention in the context of dynamic fluctuations of normal resting state connections. Chang et al. (2013) recently reported that connectivity of the anterior insula with cingulate cortex and amygdala fluctuates in parallel with heart rate variability, a measure of autonomous nervous system tone, which has also been observed to be altered in AN (Bomba et al., 2014). These within-subject variations seem to map dynamics which mirror well-known RSNs derived using patterns of spatial covariance across subjects (Taylor et al., 2012). Zalesky et al. (2014) recently observed that dynamic functional connectivity is also highly topologically structured. Hub regions, mediating inter-network crosstalk are those with the highest contribution to physiological dynamic network activity. In their study, Zalesky et al. (2014) found the angular gyrus to be one of the regions with strongest contribution to dynamic functional connectivity (the anterior insular was not a region of interest in their study). However, to verify such speculations, dynamic rs-FC analysis approaches should be applied in futures studies.

Our study has to be interpreted in the light of the following limitations. First, patients with AN are malnourished, which may lead to (pseudo)-atrophic changes of gray and white matter or altered developmental trajectories of gray and white matter maturation (Seitz et al., 2014). Increases of gray matter have also been observed in acute AN (Frank et al., 2013). Therefore we cannot exclude that detected alterations in rs-FC are also related to undernutrition. However, to avoid problems with registration of our functional data to a common coordinate system we have estimated a group-specific template during our image preprocessing (DARTEL; Ashburner, 2007). Second, we investigated a sample within an age-range hallmarked by neurodevelopmental changes. Although we could not find an association between age and the magnitude of rs-FC we cannot completely rule out that our findings are related to neurodevelopmental changes. Third, to disentangle whether brain abnormalities are cause or consequence of pathological eating more resting state studies in long-term recovered AN patients are needed. A strength of our study is the large homogenous, unmedicated sample of young acute AN patients who have had a short duration of illness. Investigating an unmedicated sample is advantageous as it allows to eliminate drug-related changes in BOLD signal (Honey and Bullmore, 2004) and brain morphometry (Moncrieff and Leo, 2010).

In conclusion, this study demonstrates increased functional connectivity within a fronto-parietal network in AN which might be related to excessive cognitive control in this patient group. Moreover we could demonstrate that the anterior insula is more strongly assigned to the DMN in acute AN which may mirror difficulties to disengage from internal mental states such as ruminations about food and bodily appearance when AN patients are not given a specific task. Both observations open up new avenues for future interventional studies. Modern psychotherapeutic approaches such as emotional acceptance behavior therapy (Wildes and Marcus, 2011) or brain stimulation and biofeedback techniques may help to balance cognitive control and self-focused ruminations via the modulation of RSN connectivity (Fox et al., 2012).

## Conflict of interest statement

In the last two years, Dr. Roessner has received payment for consulting and writing activities from Lilly, Novartis, and Shire Pharmaceuticals, lecture honoraria from Lilly, Novartis, Shire Pharmaceuticals, and Medice Pharma, and support for research from Shire and Novartis. He has carried out (and is currently carrying out) clinical trials in cooperation with the Novartis, Shire, and Otsuka companies. All other authors reported no biomedical financial interests or potential conflicts of interest.

## Footnotes

^1^www.fil.ion.ucl.ac.uk/spm/

^2^http://nipy.sourceforge.net/nipype/

^3^http:/icatb.sourceforge.net/
